# Developing the TeamOBS-vacuum-assisted delivery checklist to assess clinical performance in a vacuum-assisted delivery: a Delphi study with initial validation

**DOI:** 10.3389/fmed.2024.1330443

**Published:** 2024-02-02

**Authors:** Lise Brogaard, Kim Hinshaw, Ole Kierkegaard, Tanja Manser, Niels Uldbjerg, Lone Hvidman

**Affiliations:** ^1^Department of Obstetrics and Gynecology, Aarhus University Hospital, Aarhus, Denmark; ^2^Department of Clinical Medicine, Aarhus University, Aarhus, Denmark; ^3^Department of Obstetrics and Gynecology, Sunderland Royal Hospital, Sunderland, United Kingdom; ^4^Department of Obstetrics and Gynecology, Horsens Regional Hospital, Horsens, Denmark; ^5^Fachhochschule Nordwestschweiz (FHNW) School of Applied Psychology, University of Applied Sciences and Arts Northwestern Switzerland, Olten, Switzerland

**Keywords:** performance, emergency, obstetric, vacuum extraction, team, video, checklist

## Abstract

**Introduction:**

In Northern Europe, vacuum-assisted delivery (VAD) accounts for 6–15% of all deliveries; VAD is considered safe when conducted by adequately trained personnel. However, failed vacuum extraction can be harmful to both the mother and child. Therefore, the clinical performance in VAD must be assessed to guide learning, determine a performance benchmark, and evaluate the quality to achieve an overall high performance. We were unable to identify a pre-existing tool for evaluating the clinical performance in real-life vacuum-assisted births.

**Objective:**

We aimed to develop and validate a checklist for assessing the clinical performance in VAD.

**Methods:**

We conducted a Delphi process, described as an interactive process where experts answer questions until answers converge toward a “joint opinion” (consensus). We invited international experts as Delphi panelists and reached a consensus after four Delphi rounds, described as follows: (1) the panelists were asked to add, remove, or suggest corrections to the preliminary list of items essential for evaluating clinical performance in VAD; (2) the panelists applied weights of clinical importance on a Likert scale of 1–5 for each item; (3) each panelist revised their original scores after reviewing a summary of the other panelists’ scores and arguments; and (4) the TeamOBS-VAD was tested using videos of real-life VADs, and the Delphi panel made final adjustments and approved the checklist.

**Results:**

Twelve Delphi panelists from the UK (*n* = 3), Norway (*n* = 2), Sweden (*n* = 3), Denmark (*n* = 3), and Iceland (*n* = 1) were included. After four Delphi rounds, the Delphi panel reached a consensus on the checklist items and scores. The TeamOBS-VAD checklist was tested using 60 videos of real-life vacuum extractions. The inter-rater agreement had an intraclass correlation coefficient (ICC) of 0.73; 95% confidence interval (95% CI) of [0.58, 0.83], and that for the average of two raters was ICC 0.84 95% CI [0.73, 0.91]. The TeamOBS-VAD score was not associated with difficulties in delivery, such as the number of contractions during vacuum extraction delivery, cephalic level, rotation, and position. Failed vacuum extraction occurred in 6% of the video deliveries, but none were associated with the teams with low clinical performance scores.

**Conclusion:**

The TeamOBS-VAD checklist provides a valid and reliable evaluation of the clinical performance of vaginal-assisted vacuum extraction.

## 1 Introduction

In Northern Europe, 6–15% of women have had a vacuum-assisted delivery (VAD) (1). Notably, most births using a vacuum have good outcomes, and VAD is generally accepted as safe when performed by appropriately trained healthcare providers (2). When delivery is indicated in the second stage of labor, obstetricians must balance the differing risks of instrumental-assisted births with those of second-stage cesarean births. Women delivered successfully using a vacuum have a higher chance of uncomplicated births in subsequent pregnancies than those delivered through a cesarean section. Furthermore, cesarean birth in the second stage can be challenging because of increased maternal and perinatal risks (3). Therefore, instrumental delivery remains a core obstetrical competence, and systematic clinical performance evaluation is crucial to ensure ongoing quality assessment and research to improve clinical training (4).

Available checklists for VAD have been designed to support procedural task execution, as cognitive aids or by evaluating by item-by-item feedback. These existing checklists in vacuum extraction use simple dichotomous items (not done/done) or Likert scales (5–7). However, a growing body of empirical evidence suggests that performance assessment should include weighted checklist items to differentiate between essential and less important actions. Furthermore, the inclusion of time frames helps to create a more refined assessment of performance (8, 9). The main advantage of these performance assessment tools is the production of an objective summative score that is valuable in quality assessment, benchmarking performance and research (10).

We could not identify an existing performance assessment tool for real-life vacuum-assisted births that fulfilled the abovementioned requirements; therefore, this study aimed to develop and validate a checklist for assessing the clinical performance of VAD.

## 2 Materials and methods

### 2.1 Delphi method

**FIGURE 1:**
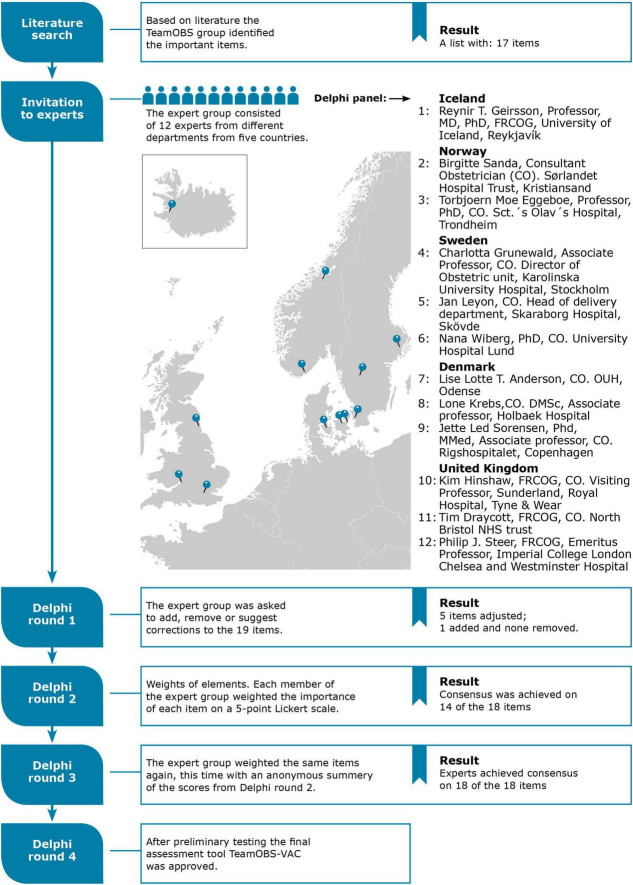
The Delphi process for the TeamOBS-VAD checklist.

We developed the TeamOBS-VAD checklist using a Delphi process to evaluate clinical performance (Figure 1). The Delphi method is an interactive process in which experts answer questions in four rounds until the answers reach a consensus. This is an internationally recognized method for solving research questions with different clinical approaches in practice and limited evidence (11–13). Our research aimed to develop a list of core items/tasks that a team should perform when conducting a VAD. The Delphi process was conducted online using Google survey tool forms.

Eighteen international obstetricians were invited to participate in the Delphi panel; 12 accepted and completed the Delphi process. The Delphi panelists were obstetric consultants from the UK (*n* = 3), Norway (*n* = 2), Sweden (*n* = 3), Denmark (*n* = 3), and Iceland (*n* = 1). The Delphi process was anonymous to ensure equal weight for all participants’ arguments and suggestions. The Delphi steering committee (NU, LB, OK, and LH) drafted a preliminary list of items identified from the literature and international guidelines, each representing a core task in the VAD.

In the first round, the panel reviewed the preliminary list of items and was asked to add, remove, or suggest the wording of items and argue why. In the second round, panelists weighted each item for clinical importance on a 5-point Likert scale, where “5” was highly important and “1” was least important. In round three, the panelists reassessed the weights after reviewing a summary of the other panelists’ scores and arguments. Consensus was defined when 90% of the panelists’ scores fell within three neighboring categories using the Likert scale range of 1–5.

The TeamOBS-VAD checklist was designed using a predefined blueprint (10). The checklist resulted in a total score calculated using a “*weighted score*” of 0–100% as a percentage of the highest possible points and the assessors’ subjective global rating, the “*patient safety score*,” ranging from 0–100% (100% served as a goal for others). The “*patient safety score*” represented the assessors’ subjective global rating of all treatment actions and offered the opportunity to evaluate aspects of performance that were not captured by the 18 items. The TeamOBS-VAD score was calculated as follows: (*weighted score* + *patient safety score*)/2.

### 2.2 Validity and reliability testing

We used the conceptual definitions and arguments for validity described by Cook et al. (14, 15) to test validity and reliability. We used video recordings of real-life VADs, collected with informed consent from all individuals present (patients and staff) in the videos from two Danish hospitals: Aarhus University and Horsens Regional Hospitals. Aarhus University Hospital, with approximately 5,000 deliveries per year, provides level III maternal care (16) and Horsens Regional Hospital, with approximately 2,000 deliveries per year, provides level II maternal care. All 17 birthing suites at the two hospitals were equipped with two or three high-definition minidome surveillance cameras and a microphone attached to the ceiling, allowing for a comprehensive view of the room. As previously described, the recordings were automatically activated using Bluetooth (17). The instruments used for vacuum extraction were a Malmström or Bird metal size 5–6 cm cup or a soft silicone cup. Videos were obtained with informed consent over 15 months between January 2015 and March 2016 and analyzed between 2018 and 2019.

Consultants LH and LA conducted tests for validity and reliability. They were experienced video raters from previous TeamOBS studies. In a session, they were trained as “raters” to develop familiarity with the checklist, followed by detailed discussions about the high and low score definitions for each item in the tool. The TeamOBS-VAD checklist was then independently applied to 60 real-life VAD video recordings. One month later, they reassessed 20% of the recordings (randomly selected) to evaluate both inter-rater and intra-rater agreements. Notably, all 60 videos were ranked based on their clinical performance scores. NU, LB, and LH reviewed the videos chronologically to determine where to set the low, acceptable, and high team performance levels.

### 2.3 Ethics

This study was approved by the Central Denmark Region’s legal department, Danish Data Protection Agency (2012-58-006), and Central Denmark Region’s Research Foundation (Case No. 1-16-02-257-14). All the participants (patients and staff) volunteered to participate and provided informed consent.

### 2.4 Statistical analysis

The clinical performance scores were analyzed on a logit-transformed scale to meet the criteria for normality and back-transformed using the inverse logit function (18). Rater agreement was described as summative using the intraclass correlation (19), Bland–Altman plots, and limits of agreement (20). STATA 17 (StataCorp LP, College Station, TX, USA) was used for statistical analysis.

## 3 Results

**FIGURE 2:**
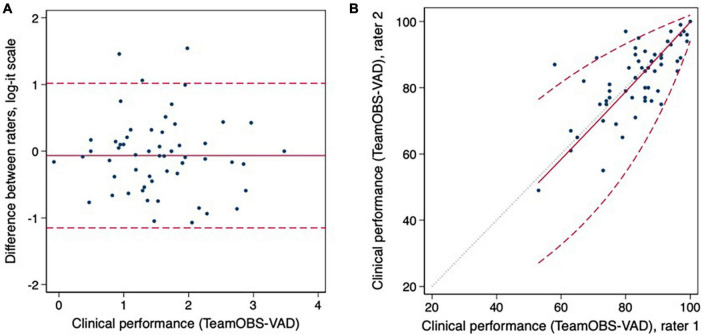
In graphs **(A,B)** the inter-rater agreement are visualized as Bland Altman plots and limits of agreement. Clinical performance data was analyzed on the log-it scale to meet the assumptions of constant mean, SD, and normality.

The Delphi panel achieved consensus in round one after adjusting for five items and adding one. Notably, all panelists weighted items according to importance on a Likert scale of 1–5; they reached a consensus on 14/18 items in the second round and a consensus on all 18 items in the next round. The TeamOBS-VAD checklist was developed and tested for usability in a simulation based on the Delphi method, and the raters found it easy to understand and use (Figure 2, Supplementary material 1).

**FIGURE 3:**
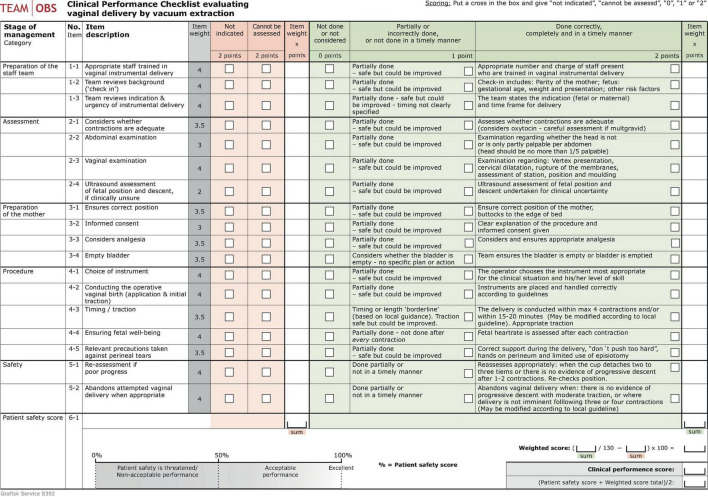
The TeamOBS-VAD checklist.

The validity and reliability were tested by applying the TeamOBS-VAD checklist to 60 videos of real-life VADs. The inter-rater agreement for an individual rater had an intraclass correlation coefficient (ICC) of 0.73; 95% confidence interval (95% CI) of [0.58, 0.83], and that for the average of two raters ICC 0.84; 95% CI [0.73, 0.91]. The intra-rater agreement was tested as raters re-evaluated 11 videos, and the agreements had an ICC 0.74; 95% CI [0.24, 0.92] for rater one and ICC 0.90; 95% CI [0.67, 0.97] for rater 2. Agreement was described using the Bland–Altman plot. The limits used to indicate low, acceptable, and high clinical performance were a score of <60%, 60–84%, and 85–100%, respectively; Figure 3. The arguments for validity and reliability are presented in Table 1.

**TABLE 1:** Arguments for validity.

Arguments for TeamOBS-VAD validity					
**Sources of validity**		**Validity question?**	**Data**	**Method**	**Results**
1.	Content evidence	Measure what it was intended to?	Delphi process	Expert panel of 12 senior obstetricians from five countries	Consensus of items, weight of importance, and final checklist
			Blueprint	Used five-step approach	TeamOBS-VAD used a rating scale with five categories, weighed items, and a global rating scale
2.	Response process evidence	Easy to use and understand?	Rater handbook	Systematic feedback Usability testing	After 1 h of training, the obstetricians were comfortable with the checklist
3.	Internal structure evidence	Distinguish high from low performance?	60 videos of real-life deliveries by vacuum	Review videos in chronological order by clinical performance, open discussion	Performance Low < 60% Acceptable 60–84% High 85–100%
		Reproducibility?	60 videos of real-life deliveries by vacuum	Inter-rater agreement Individual Average of two raters	ICC (95% CI) 0.73 [0.58, 0.83] 0.84 [0.73, 0.91]
			11 videos of real-life (18%)	Intra-rater agreement Reevaluation >1 month	rater 1: ICC 0.72 [0.24, 0.92] rater 2: ICC 0.90 [0.67, 0.97]
			Across scenarios	Indication of vacuum	Obstericians found the checklist easy to use across scenarios
4.	Relations with other variables evidence	Is the performance associated with low-risk deliveries?	60 videos of real-life deliveries by vacuum	All videos were listed for the number of contractions, position (mid, lower, outlet), and failed or successful vaginal delivery	Clinical performance had no association with the number of contractions or positions. Failed vacuum extraction was listed in 6% of the videos; however, none of these teams achieved low clinical performance
5.	Consequences evidence	Disagreements between raters	60 videos of real-life deliveries by vacuum	Disagreement >15 points between raters	Six videos (10%) Recommended two reviewers for high precision

## 4 Discussion

### 4.1 Main findings

We used an international Delphi process to develop the TeamOBS-VAD checklist to evaluate the clinical performance of VAD. The checklist allowed the calculation of the total performance score. The validity and reliability of the TeamOBS-VAD checklist were high when applied by two raters. However, they were still acceptable when used by one rater.

### 4.2 Strengths and limitations

A crucial strength of the study is the international, diverse Delphi panel as this ensured a sensible construct along with clinical applicability and increased the possibilities for international adoption and acceptability (21). The panel’s feedback and commentaries rounds guaranteed substantiated adaptations in the described tasks as well of the framework of the checklist (13). We recognize that the inclusion of a larger number of experts and inclusion of other professional groups such as midwives in expert panels could have been of additional benefit. Furthermore, as all experts in the Delphi are based Northern Europe countries, we recognize that the checklist will apply primarily in these countries. However, with minor modifications the checklist may be useful in other countries as well (22).

The use of videos of real-life vacuum deliveries was also a significant strength to the validity and reliability (15). Informed consent was obtained for all included videos, fulfilling all Danish ethical and legal requirements. However, informed consent may introduce potential selection bias, as we cannot exclude the possibility that low-performing teams were less willing to provide consent (23). It was nevertheless reassuring that 95% of the obstetricians consented to include all their videos, and only two videos were deleted when the staff withdrew consent (24).

### 4.3 Interpretation

The Delphi process was valuable in ensuring the inclusion of different international perspectives in managing vacuum extraction. The respective panelists’ national guidelines had similarities; however, some elements differed (25). Discussions in the Delphi panel included the acceptable number of pulls, when to abandon the attempt, checking the position with ultrasound (and it’s weighting according to importance), and examining and suturing the perineum after delivery. The weighted score of pain relief was discussed, as some panelists thought the idea of delivery using a vacuum without considering further medical pain relief was inappropriate. However, others did not consider the need for additional analgesia. These different views may reflect the expectations of the panelists and the availability or use of epidural and spinal analgesia (26). Two Delphi panelists preferred to include non-technical skills such as communication, leadership, and teamwork in the TeamOBS-VAD.

The steering committee had *a priori* decided not to include non-technical skills in the checklist, as validated obstetric teamwork assessment tools already exist for this purpose (27). From a methodological point of view tools for rating non-technical skills should be independent of the actual clinical problem while the clinical performance ratings need to address these specifics. Thus, the Delphi process and framework of the checklist did focus on the clinical performance (24, 28).

We did not identify any published checklists which are specifically designed to produce a summative score for VAD performance. Previously published checklists have been designed as cognitive aids, for supporting procedural task execution, or evaluating using item-by-item feedback (5–7). These classic evaluation checklists use simple dichotomous items (i.e., not done/done); however, dichotomous items are often not sufficient for the assessment of more complex tasks. Therefore, tools for performance evaluation include more categories (e.g., not indicated/incorrectly performed/performed late/timely and correctly performed) (10). Other requirements of a performance checklist include weighted checklist items to differentiate between essential and less important actions and time frames to help create a more refined assessment of performance (8, 9). Therefore, we included both weighted items and more categories and these should be considered when developing future evaluation checklists.

The summative clinical performance score is useful in (a) assessing adherence to accepted guidelines, (b) supporting individual learning by mapping the learning curve, and (c) quality assessment within a labor and delivery ward (10). We developed the TeamOBS-VAD as a tool to produce a summative clinical performance score based on items weighted for importance. We ensured that difficult deliveries and deliveries in which the teams abandoned the vaginal delivery attempt did not automatically result in a low score. Conversely, simple outlet deliveries did not automatically result in high scores. The TeamOBS-VAD clinical performance score was not associated with the number of contractions if the number was acceptable, cephalic level, rotation, or position. Failed vacuum extraction was observed in 6% of the videos; none were teams with low clinical performance scores.

Educators and trainees have experienced difficulties developing and maintaining clinical competence in VAD because of reduced working hours and instrumental delivery rates (29). Therefore, we must rethink our learning path for vacuum extraction to improve and speed up trainees’ learning. The first step could be to systematically assess performance, as improving current methods is difficult if we do not measure them objectively (30). Filming vaginal deliveries could be a second step in rethinking our learning path because a systematic assessment of the performance of trainees or departments allows us to investigate our team’s performance and offer targeted training (31). Studies evaluating video use for educational purposes have reported high patient and staff acceptability and compliance (23). Notably, 30% of the staff found that filming provoked mild anxiety; however, they confirmed that the educational value outweighed it (32). Solving the ethical and legal issues associated with video recordings in emergency care may improve our knowledge and serve as a foundation for providing better patient care (33, 34).

External validity must be considered before applying the TeamOBS-VAD checklist in other settings. Our checklist reflects adherence to guidelines and accepted practices in Northern Europe. Thus, before applying the TeamOBS-VAD checklist in different settings, it may be necessary to agree with the present statements in the tool for “done correctly.” Significant differences in opinion, the checklist, and item weights of importance will be imprecise. In addition, scoring an abandoned attempted vaginal delivery is meaningless if an urgent cesarean section is unavailable. Validity testing was conducted in two Danish hospitals, and it may be necessary to re-evaluate the validity if the delivery guidance differs substantially.

## 5 Conclusion

The TeamOBS-VAD checklist we developed is valid, reliable, and easy to use in assessing clinical vacuum deliveries. It may help train individuals and evaluate team performance in a department.

## Data availability statement

The raw data supporting the conclusions of this article will be made available by the authors, without undue reservation.

## Ethics statement

This study was approved by the Central Denmark Region’s legal department, Danish Data Protection Agency (2012-58-006), and Central Denmark Region’s Research Foundation (Case No. 1-16-02-257-14). All the participants (patients and staff) volunteered to participate and provided informed consent. The studies were conducted in accordance with the local legislation and institutional requirements. The participants provided their written informed consent to participate in this study.

## Author contributions

LB: Conceptualization, Data curation, Formal analysis, Funding acquisition, Investigation, Methodology, Project administration, Software, Validation, Writing – original draft. KH: Formal analysis, Methodology, Supervision, Writing – review & editing. OK: Conceptualization, Data curation, Funding acquisition, Resources, Supervision, Validation, Writing – review & editing. TM: Conceptualization, Methodology, Supervision, Validation, Writing – review & editing. NU: Conceptualization, Methodology, Supervision, Writing – review & editing. LH: Conceptualization, Data curation, Formal analysis, Investigation, Supervision, Validation, Visualization, Writing – review & editing.
