# Supplementary material for: Developing the TeamOBS-vacuum-assisted delivery checklist to assess clinical performance in a vacuum-assisted delivery: a Delphi study with initial validation
Source: Front Med (Lausanne). 2024 Feb 2;11:1330443. doi: 10.3389/fmed.2024.1330443 (PMC10869485; doi:10.3389/fmed.2024.1330443)
Supplement: Supplementary file 1 [file Data_Sheet_1.docx]

Supplementary Material

# Supplementary Data

**Step-by-step guide on the use of the TeamOBS-VAD checklist**

**Aim:**

To assess a team’s performance during the management of a vaginal vacuum-assisted delivery.

1. The six dimensions of a team’s performance are numbered 1 through 6 and include a total of 18 items.
2. Each of these 18 items describes a task and requires a SINGLE tick in one of the five available checkboxes (each item should have EITHER a red OR a green box ticked).
3. Red columns:
   1. The box “Not indicated” is used if the item is not indicated (e.g. in item 1-1 tick not indicated if appropriate staff are already present).
   2. The box “Cannot be assessed” in the red area is used when a task is indicated but cannot be assessed (e.g. in item 3-2 if the consultant spoke with the women, but the assessor could not hear their conversation clearly, and hence, could not evaluate whether informed consent was obtained).
   3. Each item is scored by multiplying the points by the weighting (e.g., 2 × 3.5 = 7).
4. Green columns:
   1. The boxes represent the assessment of a task “*Done correctly and in a timely manner*” (2 points), “*Done incorrectly or done correctly with delay*” (1 point), and “*Not done*” (0 point). Because time is a crucial factor in treatment, a task done correctly but with delay is assigned the same score as a task done incorrectly*.*
   2. Again, each item is scored by multiplying the points by the weighting (e.g., 2 × 3.5 = 7).
5. Calculate the sum for the green column (weighted score x points) and the sum for the red column (weighted score x points). Enter these two figures into the formula below to obtain a weighted score (minimum 0 to maximum score of 100):


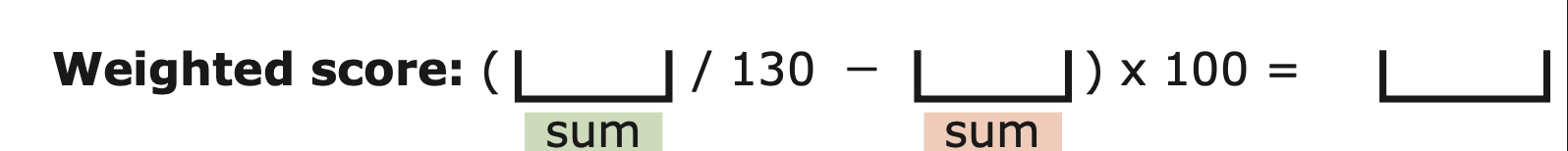


1. Next calculate the patient safety score as a continuous score ranging from 0–100%... add in a bit.
2. The final TeamOBS-PPH score is calculated as follows:


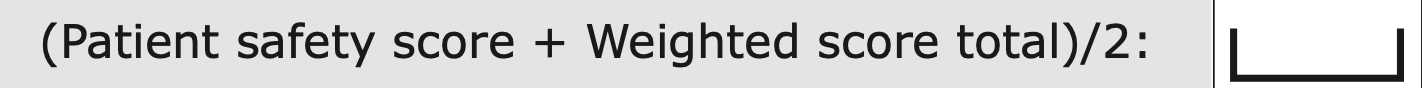


Interpretation: Low clinical performance is a score of <60%, acceptable performance 60–84%, and high performance 85–100%.
